# Developmental stages and exercise timing in relation to fear of hypoglycemia and quality of life in type 1 diabetes

**DOI:** 10.1007/s12020-026-04632-5

**Published:** 2026-05-02

**Authors:** Roberto Codella, Ambra Bisio, Daniel Gotti, Marta Bassi, Nicola Minuto, Piero Ruggeri, Livio Luzi, Davide Maggi, Emanuela Faelli

**Affiliations:** 1 Department of Endocrinology, Nutrition and Metabolic Diseases, IRCCS MultiMedica, Milan, 20138 Italy; 2 Department of Biomedical Sciences for Health, Università degli Studi di Milano, Milan, 20133 Italy; 3 Centro Polifunzionale di Scienze Motorie, University of Genoa, Genoa, 16132 Italy; 4 Department of Experimental Medicine, Section of Human Physiology, University of Genoa, Genoa, 16132 Italy; 5 Pediatric Clinic, IRCCS Istituto Giannina Gaslini, University of Genoa, Genoa, Italy; 6 IRCCS San Martino, University of Genoa, Genoa, Italy

**Keywords:** Type 1 diabetes, physical activity, quality of life, fear of hypoglycemia, exercise timing, pediatric endocrinology

## Abstract

**Purpose:**

To examine associations between physical activity (PA) characteristics and psychosocial outcomes – quality of life (QoL) and fear of hypoglycemia (FH) – in children, adolescents, and young adults with type 1 diabetes (T1D).

**Methods:**

In this cross-sectional study, 100 insulin pump-treated outpatients T1D completed 7-day PA logs capturing timing, type, intensity, and volume. QoL and FH were assessed using age-appropriate validated instruments. General linear models evaluated associations between PA characteristics and psychosocial outcomes, accounting for age group and, in sensitivity analyses, sex and HbA1c.

**Results:**

In pooled analyses (*N* = 82 complete cases), age group was significantly associated with both QoL (*p* = 0.037) and FH (*p* < 0.001), with a large effect size observed for FH. In sensitivity analyses adjusting for sex and HbA1c, the age-group effect on FH remained robust, whereas associations with QoL were attenuated. Exercise timing was associated with FH (*p* = 0.047), with higher adjusted FH scores observed among individuals reporting evening exercise. However, pairwise comparisons were not significant after correction. No significant AgeGroup×Timing interactions were detected. Preferred exercise type and intensity were not independently associated with psychosocial outcomes. In sensitivity analyses adjusted for sex and HbA1c (*N* = 62), the age-group effect on FH remained robust, whereas timing showed borderline significance.

**Conclusion:**

Developmental stage appears to be a major determinant of fear of hypoglycemia in youths with T1D. Exercise timing may contribute modestly to perceived hypoglycemia risk, particularly for evening activity, although findings were attenuated after adjustment. These cross-sectional associations highlight the importance of developmentally tailored exercise counselling, while longitudinal studies are needed to clarify directionality.

**Supplementary Information:**

The online version contains supplementary material available at 10.1007/s12020-026-04632-5.

## Introduction

Regular physical activity (PA) is a central component of type 1 diabetes (T1D) management, contributing to improved insulin sensitivity, cardiovascular health, and psychological well-being [1–6]. Barriers to participation are multifactorial, reflecting not only physiological challenges – such as glycemic variability and hypoglycemia risk – but also psychosocial concerns, particularly fear of hypoglycemia (FH) [7, 8].

FH represents one of the most persistent obstacles to exercise engagement in T1D. The unpredictability of glucose fluctuations during and after activity can generate anxiety, avoidance behaviors, and reduced enjoyment of PA [9]. Over time, heightened fear may influence exercise choices, self-management behaviors, and overall well-being. Quality of life (QoL), a multidimensional construct encompassing physical, emotional, and social domains, is closely linked to daily diabetes management demands [10]. Higher QoL has been associated with improved adherence, lower HbA1c, and reduced diabetes-related distress [11]. However, exercise-related concerns may complicate this relationship, particularly during developmental transitions characterized by increasing autonomy and responsibility for self-care [12]. Developmental stage may therefore play a critical role in shaping how young people perceive and manage exercise-related risks. Children, adolescents, and young adults differ substantially in cognitive maturity, parental involvement, risk awareness, and diabetes self-management skills. Yet relatively few studies have examined psychosocial responses to exercise across developmental strata while simultaneously accounting for exercise characteristics and clinical factors.

In parallel, physiological research has shown that specific exercise characteristics – such as timing, intensity, and modality – can influence glycemic responses in T1D. Morning exercise is often associated with lower hypoglycemia risk, potentially due to counterregulatory hormonal patterns after awakening [13], whereas evening activity may increase vulnerability to nocturnal hypoglycemia [14, 15]. Aerobic and anaerobic modalities also differ in their acute glycemic effects [14–18].

While these metabolic mechanisms are increasingly well characterized, less is known about whether such exercise characteristics are associated with perceived hypoglycemia risk or psychological well-being. Based on these physiological considerations, we hypothesized that aerobic and low-to-moderate intensity activities would be associated with more favorable psychological outcomes. Such activities generally produce more predictable glucose responses and lower risk of delayed hypoglycemia compared with high-intensity or predominantly anaerobic exercise. Greater metabolic predictability may translate into reduced fear of hypoglycemia and a greater sense of safety during physical activity, potentially supporting more positive perceptions of exercise and overall quality of life. However, empirical evidence linking specific exercise characteristics to psychosocial outcomes in individuals with type 1 diabetes remains limited.

The present study sought to examine associations between PA characteristics (timing, type, intensity, and volume) and psychosocial outcomes (QoL and FH) in a cohort of well-controlled children, adolescents, and young adults with T1D. By integrating behavioral and psychological variables within a developmentally stratified framework, this study aimed to clarify whether exercise characteristics – and particularly timing – are associated with perceived hypoglycemia risk and quality of life, beyond the influence of developmental stage.

## Materials and methods

### Study design and participants

A total of 100 outpatients with T1D were enrolled. Participants were recruited from a larger cohort of individuals with T1D attending follow-up visits at the Diabetes Clinics of the Gaslini and San Martino Hospitals in Genoa (Italy) and the San Giuseppe Hospital in Milan (Italy). All participants underwent assessment of demographic, lifestyle, and anthropometric characteristics. Body weight and height were measured to the nearest 0.1 kg and 1 mm, respectively, using a commercial scale and stadiometer (Tanita BC 571, Tanita Corporation, Tokyo, Japan). Body mass index (BMI) was calculated as weight (kg)/height (m)². Participants were asked to bring all prescription medications to the clinic visit to ensure accurate documentation. Data were collected between March 2023 and December 2024.

Inclusion criteria: patients aged > 8 years with T1D (coefficient of variation [CV] < 36% during the 3 months preceding enrolment), undergoing continuous glucose monitoring (CGM), and treated with continuous subcutaneous insulin infusion (CSII; insulin pump therapy) for ≥ 1 year. Glycemic stability was defined using CGM-derived coefficient of variation (CV < 36%), consistent with international consensus recommendations [19, 20]. Although HbA1c ≤ 7.0% is commonly considered indicative of good glycemic control [21], HbA1c was included as a covariate in sensitivity analyses rather than used as an inclusion criterion.

Exclusion criteria: pregnancy at the time of recruitment; presence of psychiatric or psychological disorders; use of β-blockers or glucocorticoids; peripheral vascular disease; peripheral neuropathy; or chronic kidney failure, including dialysis therapy.

The study protocol was approved by the Ethics Committee of the University of Genoa (protocol No. 23/2023). The study was conducted in accordance with the Declaration of Helsinki (1964) and its later amendments. Written informed consent was obtained from all participants or their legal guardians.

### Physical activity assessment and data grouping

**Fig. 1:**
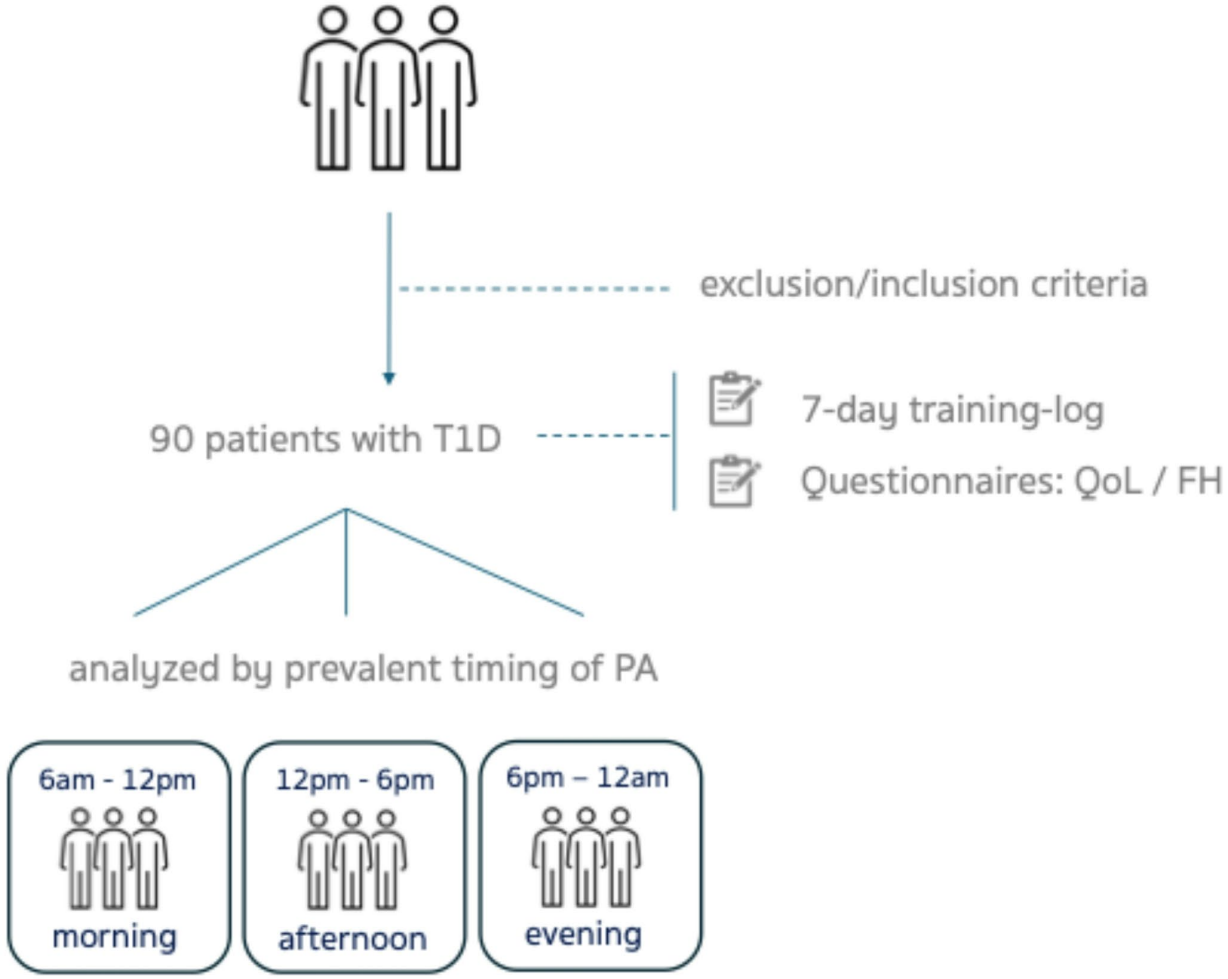
Study design

In a cross-sectional design (Fig. 1), participants were screened for all types of physical activity performed over a seven-day period in the fed state. The timing, duration, intensity, type, and volume of PA performed each day were recorded using a daily training log. The 7-day training recall is a validated method for accurately capturing PA data in both healthy and clinical populations and is considered more reliable for estimating weekly PA volume than standardized questionnaires [22]. At the time of the clinic visit, participants received a 7-day training log with detailed written instructions to ensure accurate and complete reporting. Clinicians provided additional verbal guidance on how to record activities correctly. The daily training log consisted of a structured table including predefined fields for: (1) activity type/sport performed; (2) start and end time; (3) total duration (minutes); (4) perceived intensity (light, moderate, vigorous); and (5) contextual notes (optional). Participants completed one page per day. Logs were reviewed at the follow-up visit for completeness and clarification by a clinician. A copy of the standardized log template is provided in supplementary material (Supplementary Figure A). PA volume was calculated as the product of duration (minutes) and metabolic equivalent (MET) value assigned to each activity (MET × minutes), yielding MET·min for each exercise bout. Daily PA volume was computed as the sum of MET·min accumulated across all activities performed on that day. Weekly PA volume was calculated as the sum of daily MET·min across the 7-day recording period. Participants were instructed to classify perceived intensity using simple, age-appropriate descriptors based on perceptual cues. Light intensity was defined as activity performed without noticeable increases in breathing; moderate intensity as activity causing increased breathing but still allowing conversation; and vigorous intensity as activity causing substantial increases in breathing with difficulty speaking. Each reported activity, including specifications on intensity, was converted into metabolic equivalents of task per minute (MET·min) using the Ainsworth Compendium of Physical Activities, with separate reference tables for adults and youth [23, 24]. Light-intensity activity was defined as ≤ 2.9 MET·min, moderate-intensity activity as 3.0–5.9 MET·min, and vigorous-intensity activity as ≥ 6.0 MET·min. Exercise modality (aerobic, anaerobic, or mixed) was determined based on the primary metabolic characteristics of each activity according to the Ainsworth Compendium classification and established physiological criteria. Continuous endurance activities primarily involving sustained oxidative metabolism (e.g., running, cycling, swimming) were categorized as aerobic. Short-duration, high-intensity activities predominantly relying on anaerobic glycolysis or phosphagen systems (e.g., sprinting, weightlifting) were categorized as anaerobic. Activities incorporating both endurance and intermittent high-intensity components (e.g., team sports) were categorized as mixed. For each participant, preferred exercise type was defined as the modality contributing the greatest proportion of total weekly MET·min [25]. The preferred type of physical activity/sports (aerobic, anaerobic, mixed), intensity (light, moderate, high), and preferred timing (morning, afternoon, night) were pooled among age group: under 14 (children), 14–17 (adolescents), 18–31 years old (adults). The predominant time of day at which participants accumulated the greatest PA volume was determined for each day of the week. When more than one activity was performed on the same day, differences > 10% in PA volume were used to categorize participants as *morning* (06:00–11:59), *afternoon* (12:00–17:59), or *evening/night* (18:00–23:59) exercisers for that day. Daily data were subsequently grouped according to the predominant timing of exercise (morning, afternoon, or evening). Overnight exercise bouts were excluded from analysis.

### Psychosocial assessments

Quality of Life was assessed using age-appropriate, validated questionnaires. For adults (≥ 18 years), the Diabetes Mellitus Quality of Life (DMQoL) questionnaire was used [26, 27], while for participants < 18 years the Paediatric Quality of Life Inventory (PedsQL) diabetes module was administered [28]. Both instruments evaluate multiple dimensions of QoL, including physical functioning, emotional well-being, and social functioning. For each instrument, item responses were summed and transformed into standardized relative scores according to the respective scoring manuals, with higher scores indicating better perceived quality of life.

Fear of hypoglycaemia was assessed using validated instruments corresponding to age group: the Fear of Hypoglycaemia 15-item scale (FH-15) for adults [29, 30], and the Fear of Hypoglycaemia Scale (FHS) for children and adolescents [31–33]. Both tools assess cognitive, emotional, and behavioural responses to hypoglycaemia, using Likert-type response scales. To allow comparisons across age groups despite the use of different instruments, QoL and FH scores were expressed as standardized relative scores. This approach allowed the variables to be analyzed within a unified statistical framework while preserving age-appropriate measurement tools.

All instruments have demonstrated good psychometric properties in previous studies, with reported Cronbach’s α coefficients typically exceeding 0.80 in populations with type 1 diabetes [27, 30, 32–34].

### Glycemic variables

Continuous glucose levels were monitored using the FreeStyle Libre^®^ 3 (FSL, Abbott, CA, USA) system to assess glycemic variability across the day. Several metrics were selected to describe glycemic control and variability, as well as the frequency and duration of hypo- and hyperglycemic episodes.

All variables were recorded daily over a 7-day monitoring period. Retrospective CGM data were extracted at 15-minute intervals. Daily glycemic data were then synchronized with the corresponding physical activity timepoints reported in the training logs.

### Statistical analysis

Quality of life and fear of hypoglycemia were analyzed using general linear models (GLM). QoL and FH scores were expressed as standardized relative scores to allow comparability across age groups despite the use of age-specific validated instruments. To address developmental heterogeneity, age was categorized into three strata (U14: <14 years; U18: 14–17 years; A: ≥18 years) and included as a fixed factor. Primary pooled models tested the effects of AgeGroup, exercise timing (morning/afternoon/evening), and their interaction (AgeGroup×Timing), while additionally adjusting for preferred exercise type (aerobic/anaerobic/mixed) and preferred intensity (low/medium/high). Models were fitted using ordinary least squares with Type II sums of squares. Effect sizes are reported as partial eta squared (partial η²). Where the omnibus main effect of timing was significant, Bonferroni-adjusted pairwise comparisons were performed using model-adjusted marginal means.

In sensitivity analyses, pooled general linear models were additionally adjusted for sex and HbA1c. Specifically, models included AgeGroup (U14, U18, A), exercise timing (morning/afternoon/evening), their interaction (AgeGroup×Timing), preferred exercise type (aerobic/anaerobic/mixed), preferred intensity (low/medium/high), sex, and HbA1c. Models were estimated using ordinary least squares with Type II sums of squares; effect sizes are reported as partial eta squared (partial η²). Complete-case analyses were conducted for models including HbA1c. Bonferroni-adjusted post-hoc comparisons were conducted for timing when the omnibus timing effect reached statistical significance or borderline significance.

Statistical significance was set at *p* < 0.05.

Data analysis was performed using the Statistical Package for the Social Sciences (SPSS Statistics, version 29.0.2.0., IBM Corp., Armonk, NY).

## Results

### Participants

The characteristics of the studied patients are reported in Table 1.

**Table 1:** Subjects’ characteristics

Youths with T1D (F/M)	55/45
Age (years) [min – max]	16 ± 6.6 [8–31]
BMI (kg/m^2^)	23 ± 3.4
Diabetes duration (years)	10 ± 5.8
CSII duration (years)	1.6 ± 0.18
Insulin dose (U/kg/day)	0.77 ± 0.15
HbA1c (%)	6.8 ± 1

#### Quality of life

In pooled GLM analyses (*N* = 82 complete cases), QoL differed significantly by age group (F_2,69_ = 3.47, *p* = 0.0368, partial η² = 0.091). Neither exercise timing (F_2,69_ = 1.95, *p* = 0.151), preferred exercise type (F_2,69_ = 1.83, *p* = 0.167), nor intensity (F_2,69_ = 0.20, *p* = 0.816) were significant predictors. The AgeGroup × Timing interaction was not significant (F_4,69_ = 0.21, *p* = 0.934), indicating that associations between timing and QoL did not differ across developmental strata. Adjusted marginal means were highest in adults (88.86), followed by adolescents (83.05) and children (77.37).

In sensitivity analyses additionally adjusted for sex and HbA1c (complete cases, *N* = 62; Supplementary Table S1), no predictors reached statistical significance for QoL. Exercise timing showed a borderline association (F_2,47_ = 3.04, *p* = 0.057), whereas age group, exercise type, intensity, sex, and HbA1c were not significant. The AgeGroup × Timing interaction remained non-significant (*p* = 0.961).

#### Fear of hypoglycemia

In pooled models (*N* = 82), age group demonstrated a large association with FH (F_2,69_ = 36.83, *p* < 0.001, partial η² = 0.516). Exercise timing was also significantly associated with FH (F_2,69_ = 3.19, *p* = 0.047, partial η² = 0.085), whereas preferred type (*p* = 0.146) and intensity (*p* = 0.829) were not significant. The AgeGroup × Timing interaction was not significant (F_4,69_ = 0.87, *p* = 0.484), suggesting a consistent timing pattern across developmental groups. Adjusted marginal means were highest for evening timing (10.67), compared with morning (7.45) and afternoon (7.02). Bonferroni-adjusted pairwise comparisons did not reach statistical significance, suggesting limited power for corrected contrasts. In fully adjusted sensitivity models (*N* = 62; Supplementary Table S1), age group remained strongly associated with FH (F_2,47_ = 26.65, *p* < 0.001, partial η² = 0.531). Exercise timing demonstrated a borderline association (F_2,47_ = 3.18, *p* = 0.051, partial η² = 0.119), while preferred type, preferred intensity, sex, and HbA1c were not statistically significant predictors. The AgeGroup × Timing interaction was not significant (*p* = 0.937). Model-adjusted marginal means (Supplementary Table S2) confirmed the highest FH among individuals reporting evening exercise (11.32), independent of glycemic control and sex.

## Discussion

This study examined associations between physical activity characteristics and psychosocial outcomes in children, adolescents, and young adults with type 1 diabetes. The principal finding is that developmental stage was strongly associated with fear of hypoglycemia, with a large effect size that persisted after adjustment for sex and HbA1c. In contrast, associations between specific exercise characteristics and psychosocial outcomes were comparatively modest.

Age group emerged as the most robust determinant of FH. Adults reported substantially higher FH compared with children and adolescents. This developmental gradient suggests that perceptions of hypoglycemia risk may evolve alongside increasing autonomy, responsibility for self-management, and awareness of long-term consequences. These findings underscore the importance of age-sensitive psychological support in diabetes care. Notably, the magnitude of the age-group effect on FH exceeded that of any exercise-related variable in multivariable models, suggesting that developmental context may shape perceived hypoglycemia risk more strongly than exercise characteristics themselves. This finding emphasizes that psychosocial responses to exercise cannot be interpreted independently of developmental stage.

Exercise timing demonstrated a small but statistically significant association with FH in primary analyses, with higher adjusted FH scores observed among individuals reporting evening exercise. This pattern is consistent with physiological evidence indicating increased vulnerability to nocturnal hypoglycemia following late-day activity. However, pairwise comparisons did not remain significant after correction for multiple testing, and the timing effect was attenuated in sensitivity analyses. Therefore, while evening exercise may be associated with greater perceived hypoglycemia risk, this association appears modest relative to developmental influences. Even modest timing-related differences may nevertheless influence exercise preferences and adherence over time, particularly in individuals already prone to hypoglycemia-related anxiety.

Preferred exercise type and intensity were not independently associated with either QoL or FH in multivariable models. Although physiological differences between aerobic and anaerobic modalities are well established, their psychological correlates may be more strongly influenced by individual experience, glycemic predictability, and developmental context than by exercise modality alone.

Quality of life differed by age group in primary analyses but was not robust after adjustment for sex and HbA1c. This suggests that broader developmental and clinical factors may exert greater influence on perceived well-being than specific exercise characteristics within this well-controlled cohort.

Importantly, no significant AgeGroup × Timing interactions were detected, indicating that associations between timing and FH were consistent across developmental strata rather than confined to a specific age group.

### Clinical implications

These findings suggest that psychosocial responses to exercise in T1D are shaped primarily by developmental stage. While exercise timing may contribute modestly to perceived hypoglycemia risk, individualized counseling should prioritize age-appropriate education, confidence-building strategies, and structured glucose management plans.

### Limitations

The cross-sectional design precludes causal inference. Associations may reflect reverse or bidirectional relationships; for example, higher FH may influence exercise timing choices rather than result from them. Physical activity was assessed using self-reported logs, which may introduce recall bias. Also, although device-based monitoring is considered the gold standard for quantifying movement, structured logs allow detailed capture of contextual variables – such as exercise timing, modality, and perceived intensity – that are not readily distinguishable through accelerometry alone. The chosen method reflects a pragmatic, clinic-based approach; nevertheless, future studies integrating objective monitoring with contextual self-report would strengthen measurement precision. The cohort comprised individuals with relatively good glycemic control and insulin pump therapy, potentially limiting generalizability. Additionally, complete-case sensitivity analyses reduced sample size and may have limited power to detect smaller effects.

## Conclusions

In children, adolescents, and young adults with well-controlled type 1 diabetes, developmental stage emerged as the most prominent factor associated with fear of hypoglycemia, whereas exercise timing showed modest associations and exercise modality appeared less influential than anticipated. These findings suggest that perceptions of exercise-related risk are shaped primarily by developmental context rather than by exercise characteristics alone.

Accordingly, exercise counseling in T1D may benefit from prioritizing age-specific psychological support and education alongside metabolic guidance. Prospective studies are needed to determine whether developmentally tailored interventions can reduce fear of hypoglycemia and promote sustained, confident engagement in physical activity. Understanding how developmental stage shapes perceived hypoglycemia risk may be key to fostering sustainable and psychologically secure physical activity habits in young people living with T1D.

## Supplementary Information

Below is the link to the electronic supplementary material.

Supplementary Material 1

## Data Availability

All data generated or analyzed during this study are included in this article. Further enquiries can be directed to the corresponding author.

## Declarations

### Study approval

This study protocol was reviewed and approved by the Ethics Committee of the University of Genoa, Italy, approval number 23/2023.
