# Supplementary material for: Developmental stages and exercise timing in relation to fear of hypoglycemia and quality of life in type 1 diabetes
Source: Endocrine. 2026 May 2;91(1):162. doi: 10.1007/s12020-026-04632-5 (PMC13135517; doi:10.1007/s12020-026-04632-5)
Supplement: Supplementary file 1 — Supplementary Material 1 [file 12020_2026_4632_MOESM1_ESM.docx]

# Supplementary Table S1.

Sensitivity Analysis: General Linear Models Predicting Quality of Life (QoL) and Fear of Hypoglycemia (FH), Adjusted for Age Group, Exercise Timing, Preferred Type, Preferred Intensity, Sex, and HbA1c (Complete Cases, N=62).

| Outcome | Effect | df | F | p | Partial η² |
| --- | --- | --- | --- | --- | --- |
| QoL | Age Group | 2,47 | 0.94 | 0.396 | 0.039 |
| QoL | Timing | 2,47 | 3.04 | 0.057 | 0.115 |
| QoL | Type | 2,47 | 1.54 | 0.226 | 0.061 |
| QoL | Intensity | 2,47 | 0.11 | 0.898 | 0.005 |
| QoL | Sex | 1,47 | 0.14 | 0.714 | 0.003 |
| QoL | HbA1c | 1,47 | 0.87 | 0.356 | 0.018 |
| QoL | Age Group × Timing | 4,47 | 0.15 | 0.961 | 0.013 |
| FH | Age Group | 2,47 | 26.65 | <0.001 | 0.531 |
| FH | Timing | 2,47 | 3.18 | 0.051 | 0.119 |
| FH | Type | 2,47 | 0.55 | 0.581 | 0.023 |
| FH | Intensity | 2,47 | 1.37 | 0.264 | 0.055 |
| FH | Sex | 1,47 | 1.64 | 0.206 | 0.034 |
| FH | HbA1c | 1,47 | 1.64 | 0.207 | 0.034 |
| FH | Age Group × Timing | 4,47 | 0.20 | 0.937 | 0.017 |

Note. Models estimated using Type II sums of squares. Effect sizes are reported as partial eta squared (partial η²). Complete-case analysis was used due to missing HbA1c and/or gender data.

# Supplementary Table S2.

Adjusted Marginal Means for Quality of Life (QoL) and Fear of Hypoglycemia (FH) from Fully Adjusted General Linear Models (Age Group, Timing, Preferred Type, Preferred Intensity, Sex, and HbA1c; Complete Cases, N=62).

| Factor | Level | QoL Adjusted Mean | FH Adjusted Mean |
| --- | --- | --- | --- |
| Age Group | Adults (A) | 86.22 | 18.12 |
| Age Group | Adolescents (U18) | 82.96 | 3.11 |
| Age Group | Children (U14) | 79.26 | 2.23 |
| Timing | Morning | 74.41 | 7.37 |
| Timing | Afternoon | 85.44 | 6.43 |
| Timing | Evening | 85.94 | 11.32 |

Note. Values represent model-adjusted marginal means derived from GLM with Type II sums of squares. Means are adjusted for all covariates included in the sensitivity model.

# Supplementary Figure **A**

**
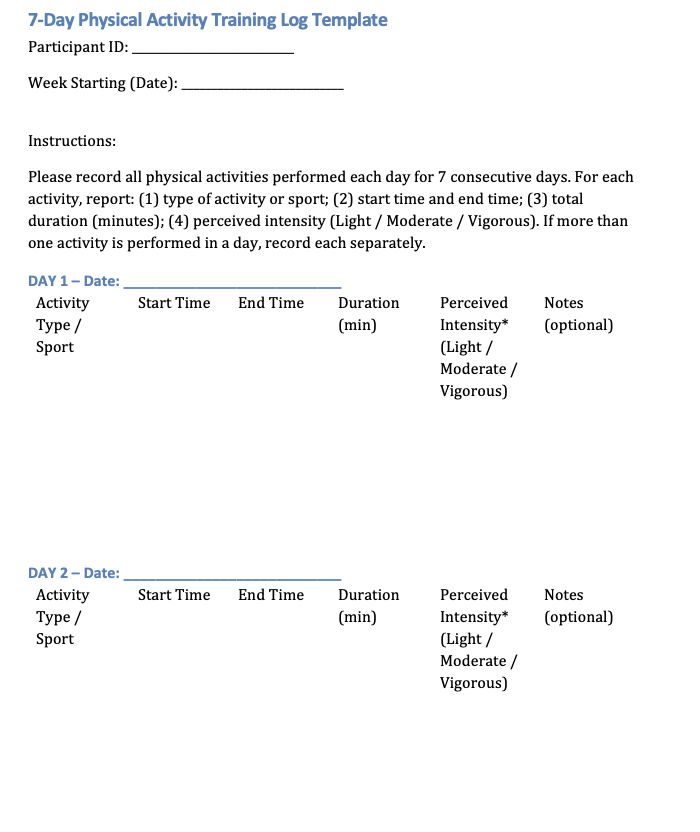
**

**
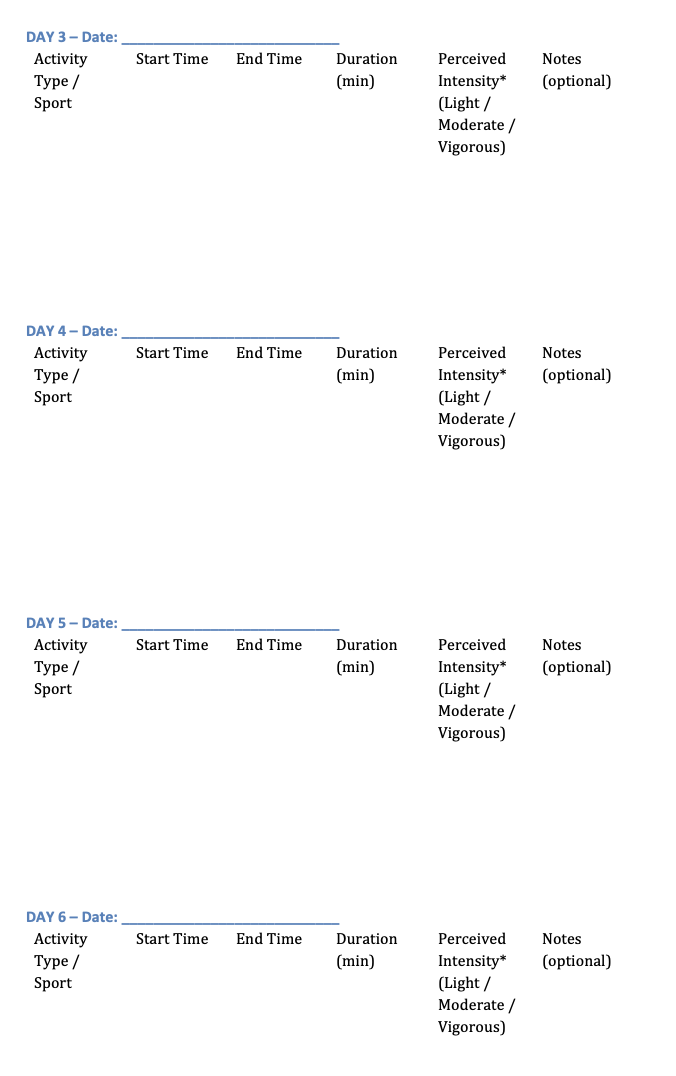
**

**
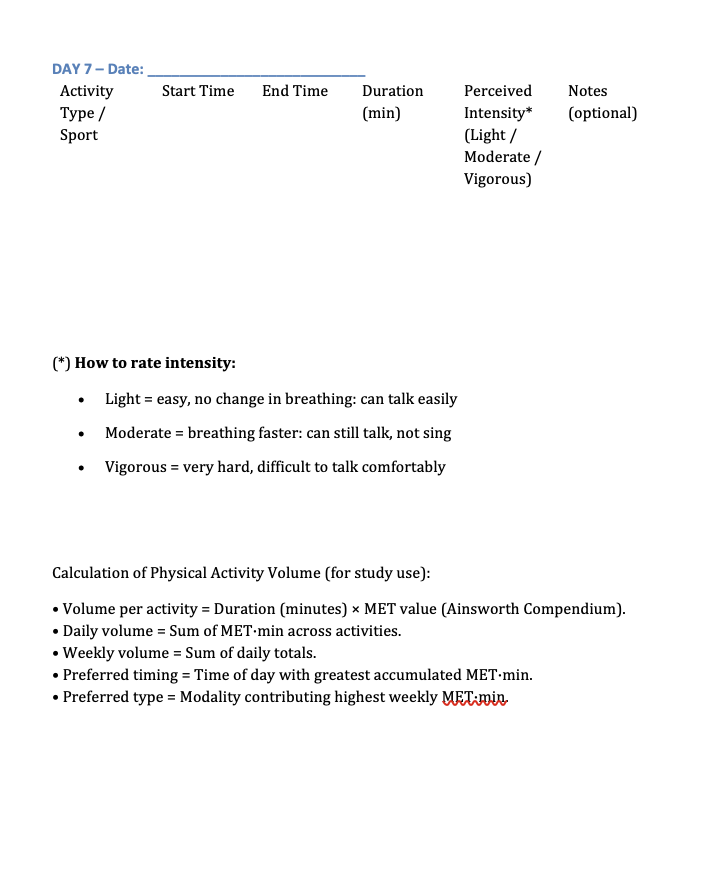
**
